# Correlation between levels of airborne endotoxin and heavy metals in subway environments in South Korea

**DOI:** 10.1038/s41598-021-95860-4

**Published:** 2021-08-24

**Authors:** Sungho Hwang, So-Yeon Kim, Sangjun Choi, Sangwon Lee, Dong-Uk Park

**Affiliations:** 1 National Cancer Control Institute, National Cancer Center, Ilsan, 10408 Republic of Korea; 2 Changwon Fatima Hospital, Changwon, 51394 Republic of Korea; 3 Department of Preventive Medicine, College of Medicine, The Catholic University of Korea, Seoul, 06591 Republic of Korea; 4 Department of Environmental Health, Korea National Open University, 86 Daehak-ro, Seoul, 03087 Republic of Korea

**Keywords:** Environmental sciences, Health occupations

## Abstract

This study aimed to evaluate the exposure levels and variation in airborne endotoxin and heavy metals such as aluminum, chromium (Cr), iron (Fe), manganese (Mn), nickel (Ni), zinc, and lead (Pb) in the three different South Korean subway environments (driver room, station office, and underground tunnel) and identify subway characteristics influencing endotoxin and heavy metals levels. Air samples were collected and analyzed using the kinetic *Limulus Amebocyte Lysate* assay and inductively coupled plasma mass spectrometers. The geometric mean was determined for endotoxin levels (0.693 EU/m^3^). It was also found that Fe (5.070 µg/m^3^) had the highest levels in subway environments while Pb (0.008 µg/m^3^) had the lowest levels. Endotoxin levels were higher in the underground tunnel and lower in the station office; the total heavy metal levels showed the same pattern with endotoxin levels. Endotoxins and total heavy metal levels were higher in the morning than at night. Positive correlations were found between endotoxin and Cr, Fe, Mn, and Ni levels. Given the correlation between airborne endotoxins and heavy metals, further studies with larger sample sizes are needed to identify the correlation between levels of airborne endotoxin and heavy metals.

## Introduction

Subway systems have been developed in many metropolises around the world. In South Korea, they have been considered as the most-used public transportation service due to its high capacity and reduced traffic congestion. Subway lines have been expanding continuously in South Korea since their inception in 1974. Most lines were built underground and has several advantages, such as saving energy, saving ground space, and efficient transit^1^.

The Indoor Air Quality Control Act was first established in 1996 in South Korea as the Underground Living Space Air Quality Control Act^2^. Since 2005, only particulate matter (PM)_10_ in subway stations is required to be monitored once a year and mandatorily reported to the Korea Ministry of Environment (KMOE)^3^. Even though monitoring systems for indoor air quality have been established and operated in underground subway stations, people are still concerned about the type and amount of air pollutants that are present in the underground environments.

Despite its significant advantages, underground subway systems have continued to have problems regarding the air quality. In particular, exposure to PMs in the underground subway systems have an adverse effect on people’s health since they are in an enclosed space with restricted ventilation^4,5^. Epidemiological studies have reported that inhalable particles can seriously damage humans’ lung function and significantly increase the risk of lung cancer. Furthermore, long-term exposure to PMs can lead to pulmonary injury^6,7^. Among airborne PMs, endotoxin, a biological agent with the pathogenicity of Gram-negative bacteria, has been implicated in the development of Gram-negative shock. Endotoxin can cause decline in lung function, respiratory inflammation, and respiratory symptoms. Additionally, a meta-analysis reported the respiratory health effects of being exposed to low levels of airborne endotoxin^8^.

Many people use the subway—including pollutant-sensitive groups such as children, medical patients, elderly people, and pregnant women—and are, therefore, exposed to the air in subway environments. People who regularly work in the subway systems are exposed to the air there for much longer periods than those who do not work in the subway systems. However, no comprehensive studies have been conducted to assess variation in airborne endotoxin and heavy metals in an underground subway environment such as the driver room of the subway train, the station office, and the underground tunnel since these areas are not commonly accessible unless subway officials are willing to cooperate and permit people to enter. The association between the levels of endotoxin and heavy metals in subway environments have never been reported, which may likely be associated with the health of commuters and people who are working in subway environments.

Current studies have mainly focused on qualitative rather than quantitative evaluation of PM pollution to help improve indoor air quality^9,10^. Thus, to the best of our knowledge, this is the first study which aimed to assess the variation in airborne endotoxin and heavy metals in underground subway environments in South Korea such as the driver room, the station office, and the underground tunnel. Furthermore, this study aimed to identify subway environment characteristics and the association between the levels of endotoxin and heavy metals such as aluminum (Al), chromium (Cr), iron (Fe), manganese (Mn), nickel (Ni), zinc (Zn), and lead (Pb).

## Materials and methods

### General information about subway environments

**Figure 1:**
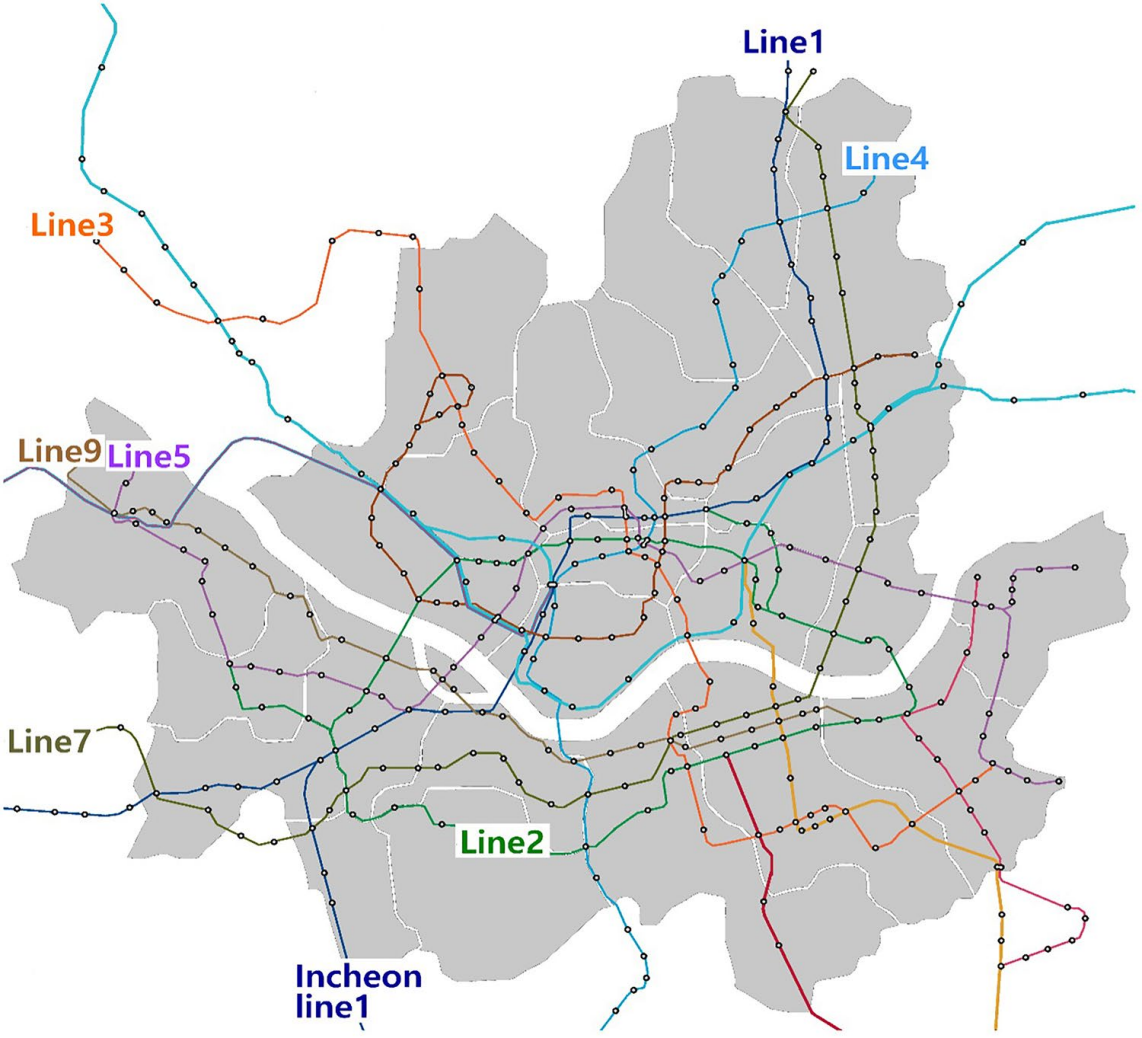
Location of subway lines 1, 2, 3, 4, 5, 7, and 9, and Incheon line 1 in Seoul and Incheon, South Korea.

A subway system in mega cities such as Seoul and the nearby Seoul area is one of the most important commuting infrastructures due to the number of passengers using a subway station from particular subway line. The general information on subway systems in metropolitan cities in South Korea is shown in Fig. 1 and Table 1. This information includes the subway line’s length, the year when the subway lines opened, and the number of stations by line. A total of 293 stations, with 270 underground and 23 above ground stations from eight lines, are currently operating nationwide. Three sampling sites—the driver room, station office, and underground tunnel—were chosen, with the cooperation of the subway officials. Drivers operate a specific subway line and are in charge of opening and closing doors through the driving room. The station office is where station management is conducted and administrative work is carried out. Underground tunnels are cleaned by technical workers who handle periodic maintenance of facilities in the tunnels. While working in tunnels, diesel engine vehicles and equipment are generally used for repairs and cleaning every day when the trains are not being operated. Several types of diesel engine vehicles are widely used for repair, maintenance, and cleaning of tunnels and other subway facilities after subway operation hours.

**Table 1:** General information on the subway line system in major metropolitan cities Seoul and Incheon.

Subway line	Length of lines (km)	Opening year of lines	Number of subway stations by line		
			Underground	Above ground	Total
1	7.8	1974	10	0	10
2	48.8	1980	37	7	44
3	38.2	1985	30	4	34
4	31.1	1985	20	6	26
5	45.2	1995	51	0	51
7	57.1	1996	49	2	51
9	40.8	2009	37	1	38
Incheon 1	30.3	1999	36	3	39
Total			270	23	293

### Sampling of endotoxin and heavy metals

We collected samples from the three sampling sites—the driver room, station office, and underground—tunnel between April and September 2018. The samples were taken at a height of 100–150 cm above the three sampling sites.

Endotoxins were collected onto glass fiber filters (37 mm diameter; SKC Inc., USA) and heavy metals (Al, Cr, Fe, Mn, Ni, Zn, and Pb) were collected onto mixed cellulose ester membrane filters (37 mm diameter; 0.8 μm pore size). The samples were preloaded into a three-piece clear plastic cassette using an air sampler (17G9 GilAir Sampler, Sensidyne, Inc., USA) at a flowrate of 2.0 L/min (± 5%) for an average of 4 h. Precautions were taken to avoid breathing on, touching, or otherwise exposing the sampling containers to human contamination while sampling airborne endotoxins and heavy metals, including the use of gloves while connecting or disconnecting the cassette and the pump^11^. After sampling, a protective covering (cap) was placed on the cassette’s inlet and outlet and the entire cassette was wrapped in its original packing and sealed with tape.

### Analysis of endotoxin and heavy metals

Endotoxin samples were stored at 4 ± 2 °C, sent to an analytical laboratory within a week of sampling with no contamination immediately upon arrival, and analyzed using kinetic-turbidimetric *Limulus Amebocyte Lysate* (LAL) assay (Associations of Cape Cod, Inc., USA). Detection and quantification of endotoxin levels were conducted using kinetic-turbidimetric LAL assay. An extraction volume of 15 mL of pyrogen-free water was added to a test tube, which was then capped and sonicated at a minimum peak frequency of 48 kHz for 1 h^11^. Thereafter, samples were centrifuged at 1000*g* for 15 min and the supernatant was transferred to a pyrogen-free test tube. An amount of 100 µL of each sample was distributed into a pyrogen-free 96-well micro-plate and incubated at 37 °C for 10 min in an automated micro-plate reader (Bio TekELx808, Bio Tek Instruments, USA)^11^. The *Escherichia coli* O55: B5 control standard endotoxin (Lonza, USA) was utilized to draw a standard curve ranging from 0.005 to 50 endotoxin unit/mL. Positive product control recoveries within 50–200% and coefficients of variation less than 10% were considered valid^11^. The endotoxin levels were expressed as endotoxin units per cubic meter of air (EU/m^3^). The assay limit of detection (LOD) was 0.01 EU/mL extract.

Heavy metal samples were stored at 4 ± 2 °C, sent to an analytical laboratory within a week of sampling with no contamination immediately upon arrival, and analyzed by the National Institute for Occupational Health and Safety 7300^12^. Pre-treatment was performed using a microwave (MARS 6, CEM, USA). The MCE filter was inserted into the microwave vessel and 3 mL of nitric acid (HNO_3_) was injected. The temperature of the sample was slowly raised to 200 °C for 15 min, and was kept inside for another 15 min. Pressure was set to 800 psi and power to 900–1050 W. Analysis of heavy metals was performed using inductively coupled plasma mass spectrometers (ICP/MS, NexION 350D, Perkin Elmer, USA). The quantities were determined by diluting each substance step by step to form a calibration curve which was drawn from the standard solution. The LOD was calculated by dipping a low concentration standard solution about seven times, referring to the standard method, and applying a value triple the standard deviation. Values below the LOD were assigned a value of LOD/$\sqrt{2}$^13^.

### Statistical analyses

Statistical analyses were conducted using SAS software, version 9.4 (SAS Institute, Inc., USA). A nonparametric analysis was performed since the endotoxin and heavy metal levels were not distributed normally or log-normally according to a Shapiro–Wilk test. Kruskal–Wallis tests were performed to determine the differences between the endotoxin, heavy metals (Al, Cr, Fe, Mn, Ni, Zn, Pb) levels and between three different sampling sites driver room, station office and underground tunnel. Mann–Whitney tests were also carried out to determine the significant difference between morning and night. In addition, Spearman’s correlation analyses were employed to examine the associations between the endotoxin and heavy metal levels.

### Ethics approval

No approval from research ethics committees was required to conduct this study.

## Results

Endotoxin levels ranged from 0.134 to 7.439 EU/m^3^ with a geometric mean (GM) of 0.693 EU/m^3^, Fe levels ranged from 0.317 to 722.384 µg/m^3^ with a GM of 5.070 µg/m^3^, which was the highest levels out of other heavy metal levels. Pb levels ranged from 0.001 to 0.284 µg/m^3^ with a GM of 0.008 µg/m^3^, which was the lowest levels out of others (Table 2). Al levels ranged from 0.104 to 18.315 µg/m^3^ with a GM of 0.506 µg/m^3^, Cr levels ranged from 0.048 to 2.491 µg/m^3^ with a GM of 0.048 µg/m^3^, Mn levels ranged from 0.005 to 7.992 µg/m^3^ with a GM of 0.072 µg/m^3^, Ni levels ranged from not detected (ND) to 0.997 µg/m^3^ with a GM of 0.032 µg/m^3^, and Zn levels ranged from 0.013 to 4.286 µg/m^3^ with a GM of 0.085 µg/m^3^.

**Table 2:** Overall levels of airborne endotoxin (unit: EU/m^3^) and heavy metals (Al, Cr, Fe, Mn, Ni, Zn, and Pb) (unit: µg/m^3^) in subway environments.

Materials	No. of samples	GM (GSD)	Min	Median	Max
Endotoxin	49	0.693 (4.3)	0.134	0.567	7.439
Al	44	0.506 (16.1)	0.104	0.440	18.315
Cr	45	0.324 (1.7)	0.048	0.262	2.491
Fe	47	5.070 (–)	0.317	4.335	722.384
Mn	47	0.072 (3.2)	0.005	0.061	7.992
Ni	47	0.032 (1.2)	<LOD^a^	0.028	0.997
Zn	42	0.085 (2.0)	0.013	0.062	4.286
Pb	44	0.008 (1.1)	0.001	0.006	0.284

^a^Limit of detection.

Airborne endotoxin and heavy metal levels were monitored in the subway driver room, station office, and underground tunnel (Table 3). Among the three different sites, endotoxin levels were highest in the underground tunnel with 1.437 EU/m^3^ (GM), and lowest in the station office with 0.392 EU/m^3^ (GM). The total heavy metal levels showed the same pattern with endotoxin levels. Although there was no significant difference (*p* > 0.05) between the three sampling points for endotoxin, the levels of the seven heavy metals were significantly different between the three places (*p* < 0.05).

**Table 3:** Levels of airborne endotoxin (unit: EU/m^3^) and heavy metals (Al, Cr, Fe, Mn, Ni, Zn, and Pb) (unit: µg/m^3^) in different indoor places in subway environments.

Materials	N^a^	Driver room			N^a^	Station office			N^a^	Underground tunnel			*p* value
		GM (GSD)	Min	Max		GM (GSD)	Min	Max		GM (GSD)	Min	Max	
Endotoxin	16	0.9219 (5.6)	0.250	7.439	22	0.392 (1.5)	0.134	1.993	11	1.437 (6.1)	0.275	6.053	0.392
Al	16	0.856 (1.9)	0.232	2.177	22	0.292 (1.6)	0.104	1.689	6	0.930 (7.5)	0.144	18.315	0.014*
Cr	16	0.414 (1.2)	0.181	0.946	22	0.173 (1.1)	0.048	0.267	7	1.318 (2.0)	0.744	2.491	0.015*
Fe	16	11.360 (2.2)	4.335	53.427	22	1.853 (4.6)	0.317	5.112	9	14.003 (9.9)	1.656	722.384	0.013*
Mn	16	0.131 (1.3)	0.042	0.873	22	0.036 (1.1)	0.005	0.300	9	0.137 (13.5)	0.017	7.992	0.013*
Ni	16	0.054 (1.0)	0.030	0.092	22	0.018 (1.0)	0.010	0.028	9	0.046 (1.4)	<LOD^b^	0.997	0.009*
Zn	16	0.079 (1.1)	0.022	0.419	22	0.066 (1.3)	0.013	0.892	4	0.448 (7.4)	0.063	4.286	0.017*
Pb	16	0.010 (1.1)	0.003	0.036	22	0.004 (1.0)	0.001	0.040	6	0.047 (1.1)	0.001	0.284	0.010*

*p < 0.05.

^a^Number of samples.

^b^Limit of detection.

**Figure 2:**
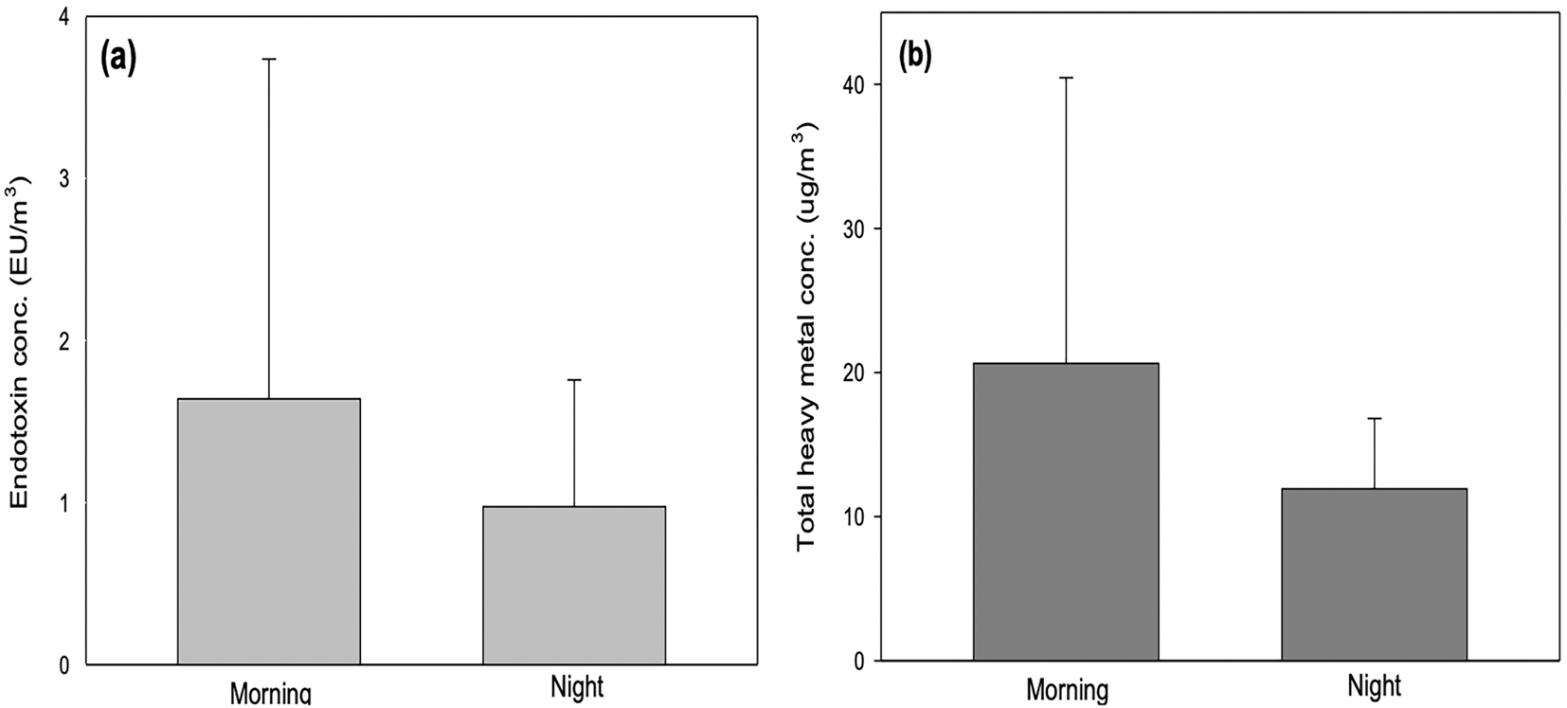
Morning and night variations in the levels of (**a**) endotoxin (*p* > 0.05), (**b**) total heavy metals (*p* > 0.05).

To evaluate time variations among these pollutants, we grouped endotoxin and total heavy metal levels by time: morning (7:30–11:00 a.m.) and night (5:00–10:20 p.m.) (Fig. 2). Endotoxin and total heavy metal levels were higher in the morning than at night (*p* > 0.05). Correlation analysis between endotoxin levels and heavy metals showed a positive association between endotoxins and Cr (r = 0.479), Fe (r = 0.441), Mn (r = 0.441), and Ni (r = 0.441) (Table 4).

**Table 4:** Correlation analysis between levels of endotoxins, Al, Cr, Fe, Mn, Ni, Zn, and Pb.

	Endotoxin	Al	Cr	Fe	Mn	Ni	Zn	Pb
Endotoxin	1.000	0.246	0.479*	0.441*	0.336*	0.571**	0.113	0.228
Al		1.000	0.474*	0.630**	0.589**	0.691**	0.406*	0.785**
Cr			1.000	0.610**	0.602**	0.780**	0.504**	0.355*
Fe				1.000	0.843**	0.800**	0.255	0.670**
Mn					1.000	0.760**	0.568**	0.731**
Ni						1.000	0.442*	0.675**
Zn							1.000	0.452*
Pb								1.000

*p < 0.05; **p < 0.001.

## Discussion

This study assessed the variations among airborne endotoxin and heavy metals, namely, Al, Cr, Fe, Mn, Ni, Cd, and Pb levels in the driver room, station office, and underground tunnel of subway stations in South Korea.

To evaluate the level of the seven heavy metals, they were compared to levels of heavy metals in a previous study conducted in Seoul, South Korea^14^. The results showed that the mean Fe level was the highest of the heavy metals in PM, followed by Zn, Ni, Mn, Cr, respectively^14^. The Fe level was consistent with that in our study, which showed that the Fe level was the highest among the heavy metals in the PM in all subway station platforms. The hexavalent Cr(VI) form of metal appears to be drastically toxic and carcinogenic; thus, it has been classified as carcinogenic to humans by the IARC^15^. The carcinogenicity of the metal targets mainly the lungs and nasal cavity^16^.

The differences between the PM levels are thought to be caused by different regional backgrounds, such as emitting sources, meteorological factors (temperature and RH), and local sources^17^. In particular, generalizing factors that may influence airborne endotoxin and heavy metal levels measured under subway systems are not easy to specify because of the subway’s characteristics, surrounding environments, and other factors including the type of subway, location, age of subway, and number of subway users^18^.

Airborne endotoxin levels were lowest in the station office, which ranged from 0.134 to 1.993 EU/m^3^ (Table 3). We found that these levels are still higher compared to that found in houses, which ranged from 0.063 to 1.720 EU/m^3^ in a previous studies on California riverside homes^19^. In the homes in an urban city of Amagasaki, Japan the levels were over tenfold higher than the maximum level, at 0.090–0.160 EU/m^3^ ^20^.

Airborne endotoxin levels were highest in the underground tunnel with a GM of 1.437 EU/m^3^ and lowest in the driver room with a GM of 0.919 EU/m^3^ (Table 3). Therefore, it can be assumed that less illumination might have affected the increase in the endotoxin levels. Based on the recommended levels of illumination for an office (400 lx) by the Korean Standards Association (KSA 3011), the endotoxin levels were significantly higher in the sampling site with 400 lx illumination^21^. Another factor affecting endotoxin levels is the number of people, which may explain why endotoxin levels, in this study, were higher in the station office than in the driver’s room. Previous studies show that endotoxin levels were significantly negatively correlated with area per person^22^, and there was a significant positive correlation between endotoxins and the number of people in a dwelling^21,23^.

According to Park et al.^11^, the size characteristics of PM is the total amount of air pollutants including endotoxin and heavy metals exposure among subway employees, maintenance workers, and subway office workers, and reported that most of the fine or ultrafine particles are assumed to stem from the use of diesel engine vehicles and heavy equipment for tunnel maintenance. Using diesel engine vehicles in semi-confined underground environments causes not only exposure to high levels of diesel engine exhaust emissions, but also an increase in PM in subway platforms and waiting rooms^24^. Another major source of PM is the widespread use of diesel-powered vehicles during maintenance of tunnels and subway facilities^24^.

Fe has the highest levels out of all heavy metal elements because iron-bearing materials are dominant (36–51% of the PM), originating from wheel/railway abrasion and the wear of iron-rich materials throughout the subway system^25^. Al levels were found to be the second highest in this study. We assumed that Al mostly come out from the steel composition of the railways^9^. According to a study conducted in Spain, the emission of abrasion products from train and rail wear in the subway system significantly contributes to increased ambient levels of elements harmful to the environment, such as Cr, Pb, and Zn^9^. Wheel/rail steel and catenary also contains a large number of additional components, namely Zn, Cr, Mn, and Ni, which are potential sources of emission contributing to the increased PM levels of these heavy metals (Fe, Cr, Zn, Mn, and Ni) in subway systems^9^.

Notably, the levels of endotoxin and Cr, Fe, Mn, and Ni are correlated (Table 4). This result is difficult to compare with other studies since there is no similar study conducted with both endotoxin and heavy metal levels. When we assume that endotoxins originate from the outer membrane of bacteria, we might explain the mechanism of this correlation between the levels of endotoxin and Cr, Fe, Mn, and Ni. Bacteria can easily attach to fine inorganic particles and be transported due to their smaller sizes. In fact, former studies have found correlations between total PM and the amount and diversity of airborne bacteria^26,27^. However, whether it will show the same patterns in long-term and large-scale sample sizes is still unknown due to the differences in sampling conditions such as temperature, relative humidity, and precipitation^28^.

The endotoxin levels measured in this study are higher in the morning than at night in the driver room; this is the same for the levels of total heavy metals (Fig. 2). We can assume that the difference in the levels of endotoxin in the morning and night were mainly due to the number of passengers who go on and off the train because the driver room’s door and window are opened when checking passengers getting on and off every single station. We found that there were more passengers in the morning than at night based on bigdata from the Seoul Metro (data.seoul.go.kr). Research on other subway systems also show that the number of passengers is positively associated with the level of culturable airborne bacteria in underground subway stations, with airborne microorganism being dispersed into the air from subway passengers’ clothing and hair^29–31^. Furthermore, preschools, kindergartens and child daycare centers, airborne endotoxin levels were also higher than in dwellings due to the activities of children and frequent diaper changes^32,33^. Wheeler et al.^34^ found that the presence of more than two people in the home increased indoor endotoxin levels in PMs, suggesting the effects of human activity. In addition, levels of airborne endotoxin were reported to be negatively associated with floor area per person at home^22^. The PM’s levels of endotoxins was higher on weekends than on weekdays due to periods of human activities and stay at home during weekends^20^.

Similar to our study, Martin et al.^35^ reported that heavy metal levels in the morning are higher than at night, but there are many factors influencing the change in the levels of heavy metals in real-time measurement due to temporal and spatial variations along the platforms, differences in the time, place, or season of the measurements, design of the stations and tunnels, variations in the train frequency, passenger densities, and ventilation systems, among other factors^35^. To reduce emissions of heavy metals in subway systems, conventional friction materials must be substituted with low metal materials such as graphite pantographs/catenaries and rubber wheels^9^.

## Conclusion

Factors affecting airborne endotoxin and heavy metal levels in three different indoor subway environments in South Korea were evaluated. Levels of endotoxin and total heavy metals were highest in the underground tunnel, followed by the driver room, then the station office. There were significantly higher levels in endotoxin and total heavy metal in the morning (7:30–11:00 a.m.) than at night (5:00–10:20 p.m.). Endotoxin levels were found to have a correlation with Cr (r = 0.479), Fe (r = 0.441), Mn (r = 0.336), and Ni (r = −0.571).

To the best of our knowledge, this is the first study to show the association between airborne endotoxin and heavy metal levels in three different subway environments in South Korea. However, there are some limitations to this study. First, the measurements of the endotoxins and heavy metals from three different places may not necessarily reflect the association with public health outcomes. Second, the short daily sampling period of about 6 h may have introduced some variation among measurements, resulting in poorer representation and weaker consistency between the levels for the entire day. Third, although endotoxin and Cr, Fe, Mn, and Ni were found to be correlated with each other, long-term evaluation for at least over a year of study using an integrated approach of quantitative exposure data in underground subway environments is needed to provide accurate assessment between levels of Cr exposure and human health. Finally, the limited sample size may not have been representative of the airborne levels of endotoxins and heavy metals, resulting in possible biases.

Despite these limitations, this study was conducted in a number of underground subway stations in areas which are not easy to access for sampling and time using standard air sampling methods. Our results are useful not only for characterizing the level of endotoxin and heavy metals in the subway environment, but also for identifying specific factors that may be significantly associated with endotoxin and heavy metals such as Cr, Fe, Mn, and Ni. Moreover, measuring the levels of endotoxins and heavy metals in the drive room, stations office, and underground tunnel is unique in this case as the study was conducted over a metropolitan area. In conclusion, although we found that there was an association between the levels of airborne endotoxin and heavy metals, these levels were lower than the threshold. Therefore, further studies with a larger sample size are needed to identify the prevalent association between the levels of endotoxin and heavy metals.

## Data Availability

The datasets generated and/or analyzed during the current study are available from the corresponding author on reasonable request.
